# Human monkeypox — After 40 years, an unintended consequence of smallpox eradication

**DOI:** 10.3389/fpubh.2022.1082586

**Published:** 2023-01-05

**Authors:** Hussain Haider Shah, Muhammad Khizer Molani, Naqiha Shabbir

**Affiliations:** Department of Internal Medicine, Dow University of Health Sciences, Karachi, Pakistan

**Keywords:** monkeypox, *Orthopoxvirus*, smallpox, smallpox vaccine, epidemic

## Abstract

Monkeypox is one of the many zoonotic viruses that belong to the *Orthopoxvirus* genus of the Poxviridae family with a similar clinical appearance to smallpox. The symptoms of monkeypox include fever, headache, muscle aches, and lymphadenopathy. The transmission of monkeypox occurs from infected animals to humans or through direct contact (sexual or skin-to-skin), respiratory droplets, and clothing such as towels. The incidence of monkeypox is rising drastically over the world. This short communication discusses the causes of the rising monkeypox cases and emphasizes strategies to prevent the spread of the virus.

Monkeypox is one of the many zoonotic viruses that belong to the *Orthopoxvirus* genus of the *Poxviridae* family, which causes a disease similar to smallpox in humans. Human monkeypox is a brick-shaped, double-stranded DNA virus that attaches to glycosaminoglycans to enter host cells. As an enveloped virus, it may enter host cells by mimicking apoptosis (1, 2). The major hosts of Poxviruses are rodents, rabbits, and non-human primates, which can occasionally be transmitted to humans facilitating the occurrence of human-to-human transmission (2). In 1958, a Danish laboratory discovered the virus in monkeys, giving rise to the term monkeypox. In 1970, a 9-month-old baby boy in the Democratic Republic of the Congo (DRC) was the first human diagnosed with monkeypox (3). The virus has now spread throughout Africa and the rest of the world. Approximately 99% of instances occur in men, and at least 95% of these patients have sexual contact with other men (4).

In a 2003 investigation of 11 confirmed cases of the monkeypox virus, prevalent signs and symptoms included headache (100 percent), fever (82 percent), sweats (82 percent), chills (82 percent), a persistent cough (73 percent), lymphadenopathy (55 percent), and sore throat (55 percent) (5). Common neurological manifestation is a prodromal headache, usually generalized or frontal, that occurs in the majority of patients. Asthenia and myalgias are also common prodromal symptoms. Neuralgia and mood disturbances can also manifest (6). According to a study, monkeypox presents as dispersed skin lesions that grow over several days. Lesions progress from papules to vesiculopustules, often with noticeable erythematous flares, and resolve with the formation of serous-to-hemorrhagic crusts that eventually detach. In patients with numerous lesions, several stages of the progression of the lesions are observed concurrently. The face, scalp, hands, arms, legs, trunk, perineum, conjunctivae, and buccal mucosa are lesion sites (7).

Monkeypox outbreak was declared a public health emergency of global concern by WHO on July 23, 2022. What began with a single, extremely uncommon case of monkeypox has already spread to many nations around the globe, with many cases having no relation to the disease's endemic regions in Central or Western Africa. The most recent estimates from the World Health Organization (WHO) as of 17^th^ November 2022 suggest that there are over 80,000 cases in 103 non-endemic countries, with approximately 29,080 confirmed cases in the United States alone and a total of 53 deaths globally (8). The emergence of monkeypox in the United States and European countries such as Spain, France, and Germany appears to be a pandemic waiting to happen because the global smallpox vaccination program was discontinued more than 40 years ago. Vaccination against smallpox can provide some cross-protective antiviral immunity against monkeypox for decades (9).

Before 1980, when the World Health Assembly declared smallpox extinct, most nations halted routine smallpox immunization (10). Because the vaccine also protects against monkeypox, the campaign has kept this disease at bay, especially in endemic regions of central and western Africa. Since smallpox vaccination ceased decades ago, the number of individuals immune to monkeypox has continuously reduced, allowing the virus to spread more easily from animals to humans and from person to person, raising the likelihood of a significant epidemic (11).

The smallpox vaccine is protective against both smallpox and monkeypox. Cross-protective immunity against West African monkeypox can be maintained for decades in the population who have received smallpox vaccination in the distant past (9). Another study demonstrates that >90% of volunteers maintain a measurable humoral or T-cell-mediated immunity for up to 75 years after smallpox vaccination (12). In another research, it was suggested that 30 years prior smallpox vaccination does not provide complete protection against systemic *Orthopoxviruses* infection (13), but vaccine-induced antibody responses were both necessary and sufficient for protection against lethal monkeypox infection (14).

Amid the COVID-19 pandemic, countries with a middle income cannot endure another epidemic due to their inadequate healthcare infrastructures and the massive pressure placed on their already overburdened healthcare systems. Insufficient numbers of healthcare experts and poor hospital facilities are inadequate to address the populace's needs. Moreover, as the monkeypox virus is rapidly spreading across countries and creating a synchronized detrimental impact, healthcare officials in countries where the virus is endemic must suggest large-scale smallpox vaccination efforts. Multiple observational studies have demonstrated that cross-immunity with smallpox vaccination is around 85 percent effective in preventing monkeypox (15). As with COVID-19, the burden of the monkeypox epidemic will not be mitigated in terms of related morbidity unless effective vaccines against the disease are created, and large smallpox vaccination campaigns are initiated in endemic and non-endemic areas. The new vaccine Tecovirimat (brand name Tpoxx) and other smallpox vaccines such as ACAM2000 and Jynneos can prevent smallpox and monkeypox with fewer adverse effects, should be stockpiled within the country, and people should be urged to get vaccinated (16). To maintain an effective surveillance system, all authorities, mainly border health services, should be instructed to strictly monitor suspected cases at all entry points, focusing on travelers arriving from African nations.

## Data availability statement

The original contributions presented in the study are included in the article/supplementary material, further inquiries can be directed to the corresponding author.

## Author contributions

HS: conceptualization, writing–original draft, final approval, and agreeing to the accuracy of the work. MM and NS: writing–original draft, final approval, and agreeing to the accuracy of the work. All authors contributed to the article and approved the submitted version.
